# The looks matter; aggression escalation from changes on phenotypic appearance in the domestic fowl

**DOI:** 10.1371/journal.pone.0188931

**Published:** 2017-12-20

**Authors:** Irene Campderrich, Guiomar Liste, Inma Estevez

**Affiliations:** 1 Neiker-Tecnalia, Department of Animal Production, Vitoria-Gasteiz, Spain; 2 IKERBASQUE, Basque Foundation for Science, Bilbao, Spain; Gaziosmanpasa University, TURKEY

## Abstract

Domestic fowl in small groups are assumed to establish hierarchical systems based on individual recognition. Conversely, interactions in large groups are modulated by badges of status. Previous studies suggested that birds differing in phenotypic appearance (PA) became targets for aggression, possibly mistaking altered PA for badges of status. We evaluated the impact of altering PA on 0, 30, 50, 70 or 100% of the birds’ house at three experimental group sizes (GS). Tested GS were 10, 20 or 40 (8 birds/m^2^, 3 pens/GSxPA, 45 total). Thus, for each GS we had groups initially homogenous (100U, U = Unmarked; 100M, M = Marked), or heterogeneous M and U phenotypes coexisting in different proportions: 30M/70U, 50M/50U, and 70M/30U, remaining unchanged until 33 weeks of age. Then, homogeneous groups (100U and 100M) were sequentially altered by marking or unmarking 30, 50 and 70% of birds at 34, 38 and 44 weeks, respectively. Aggressive interactions were observed before applying changes at 27–28 weeks (T0), and after each sequential PA change on week 35–36 (T1), 39–40 (T2) and 45–46 (T3). Frequency of aggressive interactions in altered groups at T1, T2, and T3 were compared with non-altered heterogeneous controls. Results indicate no differences across initial PA and GS treatments (T0; P>0.05). However, aggression escalation was observed at T1 immediately after the first PA manipulation (Tukey P<0.05 altered *vs* controls). Aggression in altered groups remained high at T2 when compared to controls (Tukey, P<0.05), although by T3 interactions declined to almost initial levels (Tukey, P>0.05 altered *vs* controls). Aggressive interactions at T1 and T2 were predominantly directed from un-altered towards recently altered birds, irrespectively of their initial phenotype and of the GS. These results demonstrate that a sudden change in PA affects group dynamics. Altered birds were exposed to escalated aggression even in small groups, where individual recognition was presumed.

## Introduction

The domestic fowl is a social species that when in small groups forms a stable dominance hierarchy or ´pecking order´ that is established through aggressive interactions [1–3]. Once a stable hierarchy is formed, aggressive interactions are replaced by dominance-subordinance interactions [4]. It is assumed that under this type of hierarchy birds recognize group mates individually [5] and remember the outcome of aggressive encounters [6].

The frequency and intensity of aggressive interactions to form a stable hierarchy are group size dependent [5, 7–9], as higher number of interactions among group members would be required to establish dominance relationships in larger groups. In addition, remembering the outcomes of all occurring interactions within a large group can be challenging, leading to less stable social structures. Similarly, increased aggression during hierarchy formation seems to occur only when group sizes remain relatively small, while in larger groups aggressive interactions are lower than expected [10–12]. These results led to consider that domestic fowl living in large groups were likely to base their social relationships in a more flexible, tolerant system [10, 13, 14].

With regard to negative impact on poultry production, the most conflicting group size appears to be intermediate sized groups, as opposed to larger groups (60 or 120) as described by Keeling et al, [15]. These authors proposed that group sizes around 30 birds could represent the turning point between establishing a hierarchical system typical of small group sizes [16, 17], to a tolerant social system better suited for large group sizes. Pagel and Dawkins [18] provided the mathematical frame to explain this social plasticity by showing that trying to form a hierarchy would only be cost effective in a narrow range of (low) group sizes. In large groups, where individual recognition is not feasible, social interactions would be modulated through badges of status [18].

Although, olfactory and auditory cues may help individuals in social contexts [19–21] social discrimination in the domestic fowl seem to be based on visual cues [22, 23]. In fact, visual cues such as comb and wattle size and colour, body size or plumage colour, all are known to provide important information regarding the health status [24], fighting abilities and competitive potential of the domestic fowl [25–29]. Among chickens, and numerous wild bird species such as house finches (*Carpodacus mexicanus)* or Eurasian siskins (*Carduelis spinus*), status signals are often presented as ornamental traits located around the head and neck area [24, 30, 31] which highlights the relevance of this body area in the assessment of social contests. Considering that domestic fowl use visual cues to assess individuals’ competitive ability, it is not surprising that aggression is generally directed towards individuals presenting a discrepancy from the flock ´normal´ phenotypic appearance, including changes in plumage coloration [14, 32, 33].

Other possible explanation may involve more complex evolutionary processes like kin selection [34, 35] or, alternatively, phenotype matching [36]. It is speculated that phenotypic appearance may serve to identify the degree of kinship and would explain why individuals with similar appearance would tend to cooperate and interact less aggressively [34, 35, 37]. However, discrimination may also arise through a more parsimonious mechanism, phenotype matching. Phenotype matching would permit group-member and species recognition [36, 38] by learning the phenotype of familiar relatives, or of oneself (self-referent phenotype matching). Through this mechanism, animals would form a phenotypic template to compare against the phenotypes of familiar and unfamiliar individuals [39, 40]. Phenotype matching may also help recognizing unhealthy individuals that can bring a significant risk to the population [24, 41]. Thus, phenotype templates would facilitate the identification of unrelated individuals that could out-compete locals for valuable resources, or of sick ones that could be vectors of diseases to the local population.

Phenotypic templates are formed early during post-natal development in precocial birds, based on the existing phenotypes in their social environment [40]. When more than one phenotype co-exist, it is logical to expect that birds would tend to identify themselves with the most common phenotype in the group. It could be speculated that diversity in phenotypic appearance occurring early in life would be easy to incorporate into the group social dynamics. Contrarily, the emergence of new phenotypes in adulthood, once the template is well-established, would be expected to be much more disruptive.

Commercial flocks of domestic fowl such as laying hens have been genetically selected for performance and homogeneity in a wide range of parameters (body weight, feather colour, sexual maturity, eggshell colour, egg weight [42]) and management practices are design to maintain bird homogeneity. However, phenotypic variability may emerge during the production cycle as a result of individual differences on development, feed intake, health status or injuries, among other factors. In alternative production systems phenotypic variability may be due to the use of mixed lines to maintain local breeds, or to offer a wider variety of products (e. g. white and brown eggs [43]). Thus, phenotypic variability may be relevant for the welfare, health and performance of these flocks.

In a previous study we investigated the effects of altering the phenotypic appearance (PA) of different proportions of birds (0, 30, 50, 70, 100% of birds altered) upon arrival to the experimental facility at one day of age [44, 45]. The birds were maintained at three experimental group sizes (GS; 10, 20 and 40 individuals) during the rearing period. In these studies a larger number of social interactions, aggressive and affiliative, were found in small groups of 10 compared to groups of 40 [45], while locomotion was higher in larger group [44]. By contrast, the effects of PA were unclear, although there was some indication that aggression was mainly directed from unmarked (U) towards marked (M) birds irrespective of the proportion of U or M individuals in each group [45].

In this follow up study we investigated the impact of manipulating the phenotypic appearance in adult domestic fowl (Hy-Line Brown). We hypothesized that manipulation of the phenotypic appearance in socially stable groups of adult birds will produce a sudden increment (escalation) on aggressive interactions. We predicted that the impact of such manipulation will be smaller in small groups where individual recognition is assumed (e.g. 10 birds) as compared to larger groups where individual recognition is less likely. It was also predicted that the response to the alteration of the phenotype will be stronger the lower the proportion of altered birds and that aggression will be specifically directed towards recently altered birds.

## Material and methods

This project was approved by the Ethical Committee at Neiker-Tecnalia and the Livestock Services of the Regional Government (Diputación Foral de Alava, permit number CEE_2010_002), complying with the Spanish legislation regarding the use of animals for experimental and other scientific purposes (Real Decreto 1201/2005). This study was part of a larger project that evaluated different aspects of phenotypic appearance and group size on the welfare, health and performance of laying hens.

### Animals and housing conditions

1050 one day old laser beak-trimmed female chicks of a laying strain (Hy-Line Brown) were obtained from a commercial hatchery (Avigan-Terralta, Tarragona, Spain). They were transported to the experimental poultry facility in Neiker-Tecnalia (Vitoria-Gasteiz, Spain). The facility contained 45 experimental pens that were constructed with PVC piping and plastic netting. Pen walls were shielded with a dark plastic to prevent visual contact across pens. Pen floors were covered with 1.5 Kg/m^2^ of wood shavings. Drinking (1 nipple drinker/5 birds) and feeding space (4cm/bird) in each pen was proportional to the number of birds housed. Birds were fed *ad libitum* with a commercial diet according to their rearing stage. Lighting, temperature and ventilation were controlled with a computerized system and followed standard commercial practices. Ambient temperature at arrival of the chicks was 36ºC and was progressively decreased according to standard management practices until reaching 18-20ºC at six weeks of age. After that, temperature was maintained through the study. The lighting programme was also standard; 24 h of light provided the day of arrival which was progressively reduce to reach 9h at 14 weeks of age. Photo-stimulation started at 15^th^ weeks to reach 16h light/8 h dark at the onset of lay (first egg laid 16^th^ week of age). This photoperiod was maintained during the experiment (27 to 46 weeks). At 14 weeks of age, before the onset of lay, pens were furnished with nests and perches according to national legislation (Directive 1999/74/CE, Real Decreto 3/2002).

### Experimental design

Preliminary studies were conducted with these birds during their rearing phase [44, 45]. During these initial studies, one day old chicks were randomly allocated to one of the 45 experimental pens housing 10, 20 or 40 birds (N = 15 pens per GS). In order to maintain a constant density (8 birds/m^2^), pen sizes were adjusted to GS: 0.75 x1.78 m (1.25 m^2^), 1.00 x 2.50 m (2.5 m^2^) and 2.00 x 2.50 m (5 m^2^), for GS 10, 20 and 40 birds, respectively. GS treatments were combined in a full factorial set up with 5 different initial phenotypic appearance (PA) treatments. The PA treatments consisted on the manipulation of the appearance of different proportions of birds within each group (0, 30, 50, 70 or 100%, N = 9 for each PA treatment). Consequently, two types of groups were formed: homogeneous populations were all group members were either unmarked (100U) or marked (100M) and heterogeneous populations were M and U birds coexisted in the same pen but at different proportions (30M/70U, 50M/50U, 70M/30U). Each GS by PA combination treatments were replicated in 3 pens.

The PA alteration consisted of a black mark made with a non-toxic dye that covered the feathers on the back of the birds´ head [14, 32]; see S1 Fig. To maintain PA treatments during growth, marks were reapplied as needed (every 3 to 6 weeks, up to 20 weeks of age). Marks were made as similar as possible and were always performed by the same team that agreed in the location and area covered that was proportionate to the growth of the birds. In addition, each bird was individually identified by two laminated paper tags attached to the sides of the neck (S1 Fig) following procedures as described in Cornetto and Estevez [46]. The tags included the pen number and the bird ID (for further details see Campderrich et al. [45]). These tags were displayed by all birds (both M and U) so their effects on PA were standardized. Additionally, previous research found that pecking at the tags decreased to negligible levels after the first week due to habituation [32] suggesting that the tagging did not interfere with the effects of the PA treatments applied.

The birds remained under the above experimental conditions until the onset of the current phase of the study, when manipulations over homogeneous groups (100M and 100U) took place on the adult birds. The first PA alteration took place at 34 weeks of age by randomly marking (100U) or unmarking (100M) 30% of the birds per pen. The marking was performed as explained above. Unmarking was achieved by applying an H_2_O_2_ solution to the originally black coloured feathers [47], returning them to their natural brown coloration. After this procedure, 100U groups changed to 30M/70U (30M being the recently altered subgroup), while 100M groups were converted into 70M/30U (30U being the recently altered subgroup). The second PA change was applied at 38 weeks, with an additional alteration to 20% of the birds per pen. This resulted in pens with a 50M/50U composition: half of them where 50M were originated by marking from 100U, and the other half where 50U resulted by unmarking from 100M. Finally, the 3^rd^ PA change was applied at 44 weeks where an extra 30% of birds per pen were altered. This resulted in the final groups of 30U/70M (originally 100U) and 30M/70U (originally 100M). See Table 1 for a detailed description of the experimental design.

**Table 1:** Experimental design. Three different group sizes (GS) were tested (10, 20 and 40) for each original phenotypic appearance (PA) treatment: 100% U (100U), 30% (30M/70U), 50% (50M/50U), 70% (70M/30U), 100% M (100M)). U: Unmarked, M: Marked. Originally heterogeneous groups: 30, 50 and 70% altered from day one were used as controls. Adapted from Marin et al. [47].

		Weeks of age (obsevation period)				
		27–28 (T0)	35–36 (T1)	39–40 (T2)	45–46 (T3)	
	*Group Size*	*Original groups*	*1st PA change (30% altered)*	*2nd PA change (50% altered)*	*3rd PA change (70% altered)*	# Pens
Homogeneous Groups (Sequentially altered)	10	0% Unmarked	30%Marked/ 70% Unmarked	50% Marked/ 50% Unmarked	70% Marked/ 30% Unmarked	3
	20					3
	40					3
	10	100% Marked	70% Marked/ 30% Unmarked	50% Marked/ 50% Unmarked	30% Marked/ 70% Unmarked	3
	20					3
	40					3
Heterogeneous Groups (Controls)	10	30% Marked/ 70% Unmarked	30% Marked/ 70% Unmarked		30% Marked/ 70% Unmarked	3
	20					3
	40					3
	10	50% Marked/ 50% Unmarked		50% Marked/ 50% Unmarked		3
	20					3
	40					3
	10	70% Marked/ 30% Unmarked	70% Marked/ 30% Unmarked		70% Marked/ 30% Unmarked	3
	20					3
	40					3
		45 pens observed	36 pens observed	27 pens observed	36 pens observed	45

### Data collection

Direct behavioural observations of each pen were carried out by the same observer between 8:30 and 14:00. The observations were conducted during two consecutive weeks for each time period, starting prior to the birds´ manipulation (T0, weeks 27–28). Then, observations took place after each PA change at T1 (weeks 35–36), T2 (weeks 39–40) and T3 (weeks 45–46). Once the changes were introduced in homogeneous populations we waited for three days before starting observing the birds.

During the first set of observations (T0) all 45 pens were observed to determine the basal levels of aggressive interactions occurring in original groups. The number of pens observed afterwards varied as we focused on collecting data from the recently altered groups (originally 100M and 100U) and their corresponding controls. Thus, after the first PA alteration (T1) we observed a total of 36 pens, corresponding to 30M/70U and 70M/30U groups originated from initially homogeneous groups and their controls. For T2 a total of 27 50M/50U pens were observed (recently altered and controls). Finally, 36 pens were observed after the third PA alteration (T3) corresponding to 30M/70U and 70U/30M (recently altered and controls). See Table 1 for a summary of the treatments and comparisons performed at each age period.

For each time period (T0 to T3) 10 min direct continuous behavioural observations were collected four times for each pen (40 min total observation per pen) in two weeks period. Pen order observation was randomized. During data collection the same observer sat quietly outside the pen and waited until the birds resume normal activity before starting behavioural observations. The birds were habituated to the regular presence of observers from one day old as they had participated in a previous lengthy behavioural study. All aggressive interactions, fights, threats, aggressive pecks, chases and leaps (according to Estevez et al. [13], see ethogram Table 2) were recorded. The Observer software (V 10.0, Noldus) was used to collect data from each interaction, including phenotypes and IDs´ of the specific individuals interacting. However, when recording the birds´ ID was not possible, due to the bird´s position or in the rare event of several interactions occurring simultaneously, at least the phenotypes of the interacting pair were always recorded. Thus, it was possible to calculate the frequency of interactions between the different phenotypes in each pen, with four possible combinations: MM from marked to marked; MU from marked to unmarked; UM form unmarked to marked; UU from unmarked to unmarked).

**Table 2:** Ethogram defining the aggressive interactions recorded: Aggressive pecks, chases, leaps, threats and fights.

Ethogram for aggressive interactions (Adapted from Estevez et al. 2002 [13])	
**Aggressive peck** (event)	The bird raises its head and vigorously stabs its beak towards another bird (usually directed to the head and neck region).
**Chase** (event)	The bird runs after another bird for at least three steps in an aggressive manner.
**Leap** (event)	The bird jumps and kicks its feet forward towards another bird.
**Threat** (event)	The bird stands with head clearly raised (sometimes accompanied with raising of the neck feathers) in front of another bird who held its head at a lower level.
**Fight** (event)	Two birds stand in front of each other threating and delivering pecks to each other in rapid succession, sometimes accompanied by jumps. Peaks, leaps and threats occurring during a fight sequence were not recorded independently.

### Statistical analyses

Due to the low incidence of aggression observed at T0, all aggressive interactions (fights, threats, aggressive pecks, chases and leaps) per pen and time period were lumped into one category called total aggression. Total aggression per pen was standardized according to GS, to allow for statistical comparison among groups of different sizes. The resulting data set was analysed using linear mixed models (PROC MIXED) with GS and PA and their interactions as fixed factors and pen as random effect. Sequential phenotype alteration led to different proportions of PA treatments through time, so each time period was analysed separately. Data were log+1 transformed to meet normality and homoscedasticity assumptions. Significant differences across treatments were further analysed using Tukey post-hoc comparisons.

A second set of analyses was performed (always using data standardized according to GS) to determine the changes in aggression levels occurring across time for each specific PA treatment. Linear mixed models were built including GS as fixed effect and time period as repeated measure. In this case a square root transformation was applied to meet the assumptions of normality and homoscedasticity. Tukey post-hoc comparisons were again employed to clarify significant differences across time periods.

A third set of analyses were conducted to evaluate the directionality of the aggressive interactions in each GS and PA treatment. The observed frequency of aggressive interactions per pen and time period was calculated for each possible interacting pair (MM, MU, UM, UU). We then calculated the expected frequency of aggressive interactions per pen and time period, for each possible interacting pair, assuming that aggressive interactions occurred at random. Expected values were calculated considering the frequency of each phenotype in the pen and the interacting probabilities for each possible pair. Lastly, we calculated the difference between observed and expected frequencies. Significantly higher/lower observed than expected values for a particular interacting pair, would demonstrate the directionality of the aggressive interactions. An independent analysis was conducted for each time period (T0 to T3) to compare recently altered groups with their corresponding controls. The linear model used included GS, PA, type of interacting pair and their interactions as fixed effects. However, when non-significant interactions were detected they were removed from the model one by one according to their AICC. Data were log+1 transformed to meet normality assumptions and Tukey post-hoc comparisons were used to detect differences across treatments. All statistical analyses were conducted using SAS 9.3 software package (SAS Institute, Cary, NC, USA).

## Results

**Fig 1:**
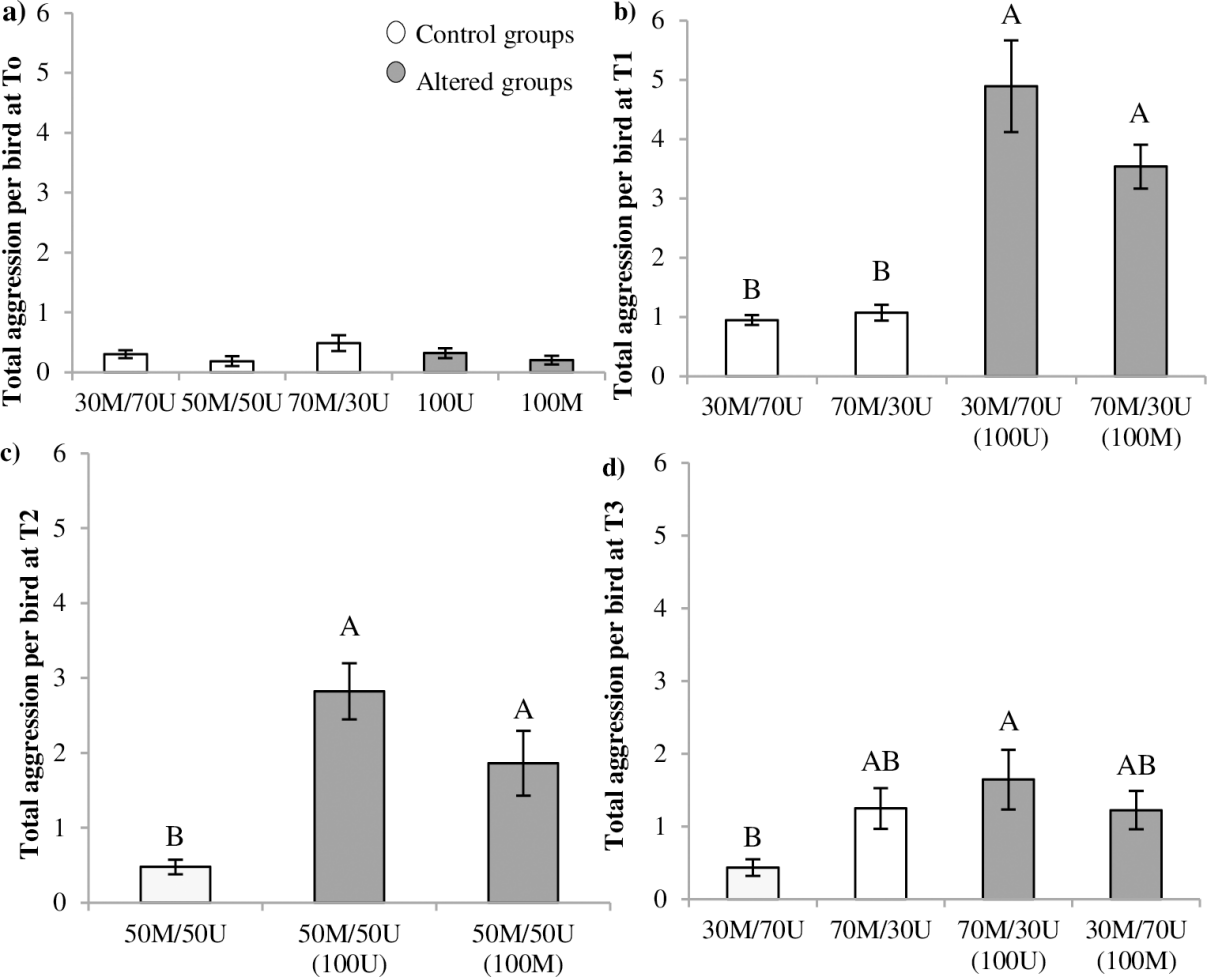
Total aggression (interactions per bird/40 min). Frequency of total aggression per bird at T0 (27–28 weeks; 1A), T1 (35–36 weeks; 1B), T2 (39–40 weeks; 1C) and T3 (45–46 weeks, 1D). Bars represent means ± SE. M = marked; U = unmarked. Phenotypic appearance (PA) treatments: originally homogeneous (100U, 100M) and controls (30M/70U, 50M/50U, 70M/30U). Different letters denote significant differences among PA treatments at *P*<0.05.

The frequency of total aggressive interactions at the onset of the study (T0) was low and similar for all PA (*F*_4,30_ = 1.63, *P* = 0.19) and GS treatments (*F*_2,30_ = 0.87, *P* = 0.43) or their interaction (*F*_8,30_ = 0.59, *P* = 0.77; Fig 1A). At T1, after the 1^st^ PA change was applied to originally homogeneous groups, total aggression showed a 3 to 4 fold increment in recently altered as compared to control groups (*F*_3,24_ = 44.17, *P*<0.0001; Fig 1B). These differences decreased but were still evident at T2 (*F*_2,18_ = 15.74, *P* = 0.0001; Fig 1C). As the proportion of recently altered birds increased by T3, total aggression receded to similar levels as controls. Regarding this last PA change (T3), the main effect of PA still showed statistical significance (*F*_3,24_ = 3.95, *P* = 0.02; Fig 1D), but post hoc comparisons revealed no differences among recently altered and control groups.

GS did not affect total aggression per bird until T3 (*F*_3,24_ = 3.94, *P* = 0.02), when birds in GS40 showed higher levels of total aggression than GS10 (Tukey *P*<0.05; 0.69±0.24a, 1.14±0.28ab, 1.58±0.24b; mean ± SE for GS 10, 20 and 40 respectively). The interaction GS by PA did not affect the total aggression per bird (*P*>0.05).

**Fig 2:**
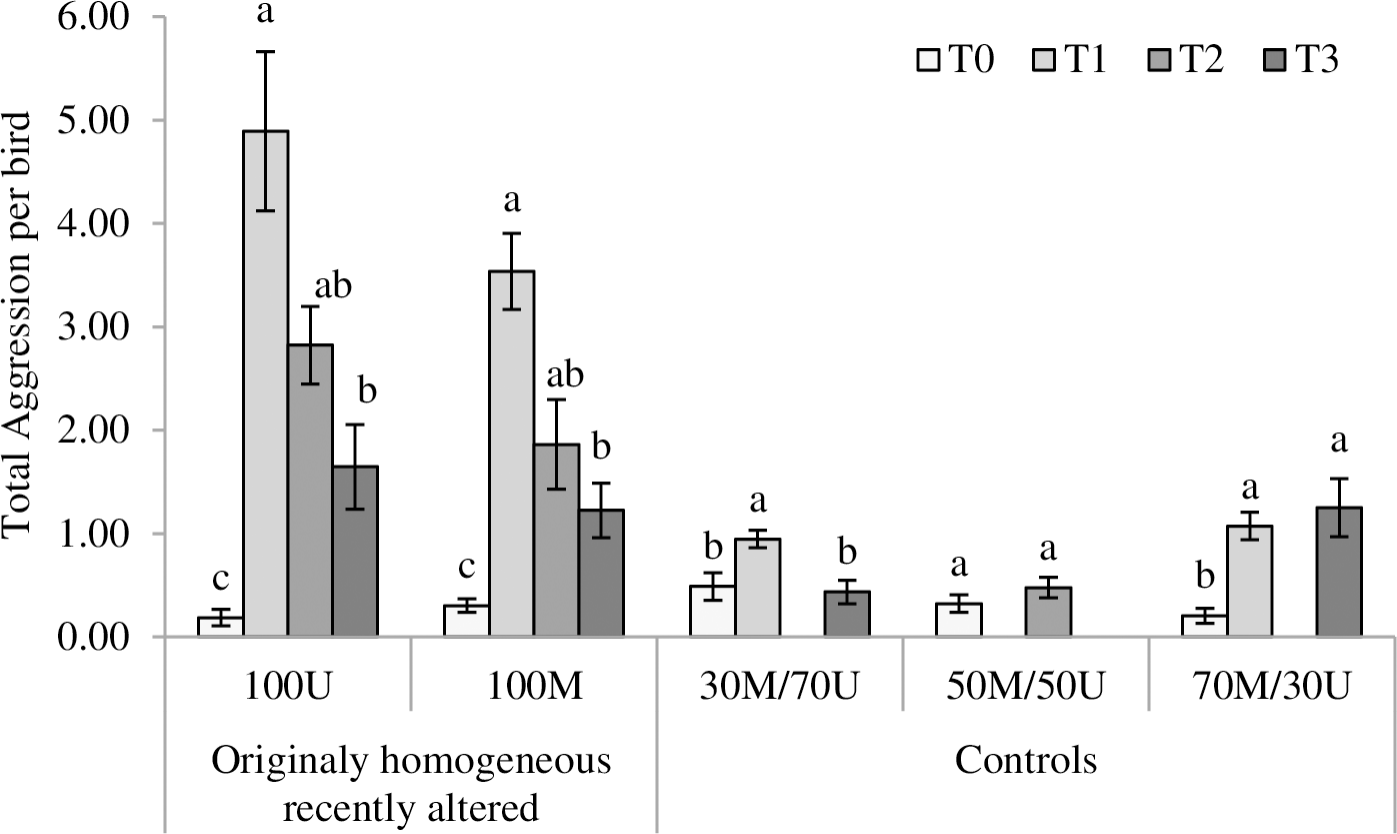
Changes in total aggression (interactions per bird/40 minutes) for each phenotypic appearance (PA) treatment across time. Changes in total aggression per bird for each PA treatment; originally homogeneous groups (100U, 100M) and controls (30M/70U, 50M/50U, 70M/30U), across time periods: T0 (27–28 weeks), T1 (35–36 weeks), T2 (39–40 weeks) and T3 (45–46 weeks). Different letters denote significant differences across time (*P*<0.05).

Our second set of analyses showed the impact of sequentially altering PA treatments through T0 to T3 (100M: *F*_3,18_ = 33.43, *P*<0.0001; 100U: *F*_3,18_ = 33.33, *P*<0.0001; Fig 2). Total aggression per bird increased in 30M/70U and 70M/30U control groups at T1, and in 70M/30U control groups at T3, as compared to T0 (30M/70U time effect *F*_2,12_ = 11.6, *P* = 0.002; 70M/30U time effect *F*_2,12_ = 21.5, *P* = 0.0001, respectively Fig 2). This increase occurred even though PA was not altered in these groups. No changes in total aggression were observed for 50M/50U (*F*_1,6_ = 1.33, *P* = 0.29; Fig 2).

Interestingly, GS did not affect total aggression through the PA changes applied across time (*P*>0.05, all cases). A GS effect was detected only for 30M/70U control groups (*F*_2,6_ = 5.43, *P* = 0.045), where GS20 showed higher total aggression than GS10 (0.83±0.11 and 0.43±0.15, respectively, GS40 showed intermediate values 0.6±0.11). The interaction GS by time period did not affect the total aggression per bird (*P*>0.05).

**Fig 3:**
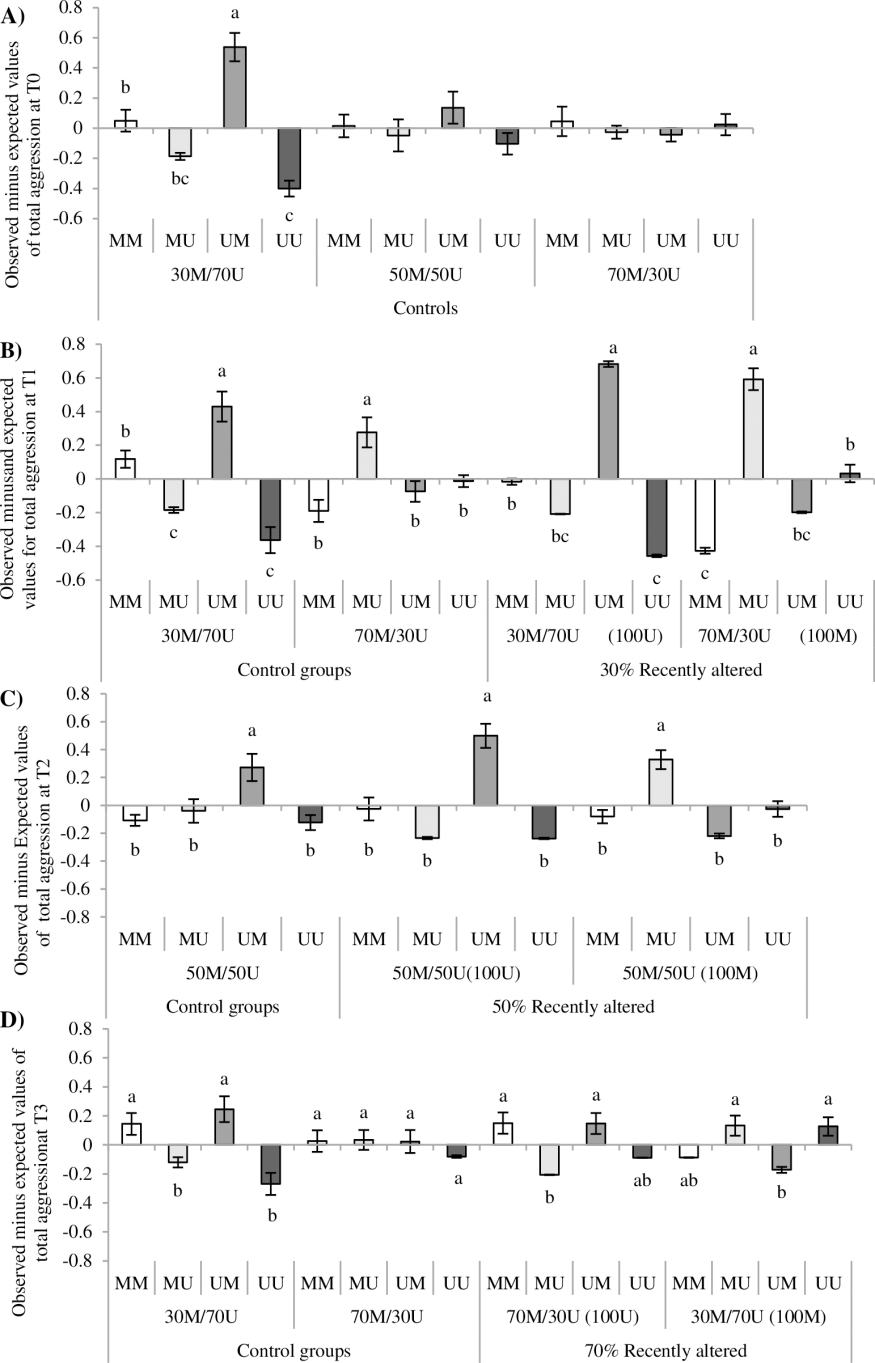
Directionality of aggressive interactions across time. M = marked; U = unmarked. Differences between observed and expected aggressive interactions (means ± SE) for each possible interacting pair (MM, MU, UM and UU) and phenotypic appearance (PA) treatment: originally homogeneous (100U, 100M), and controls (30M/70U, 50M/50U, 70M/30U). 3A) T0: 27–28 weeks; 3B) T1: 35–36; 3C) T2:39–40; 3D) T3:45–46. Different letters indicate significant differences among interacting pairs within the same PA treatment.

**Fig 4:**
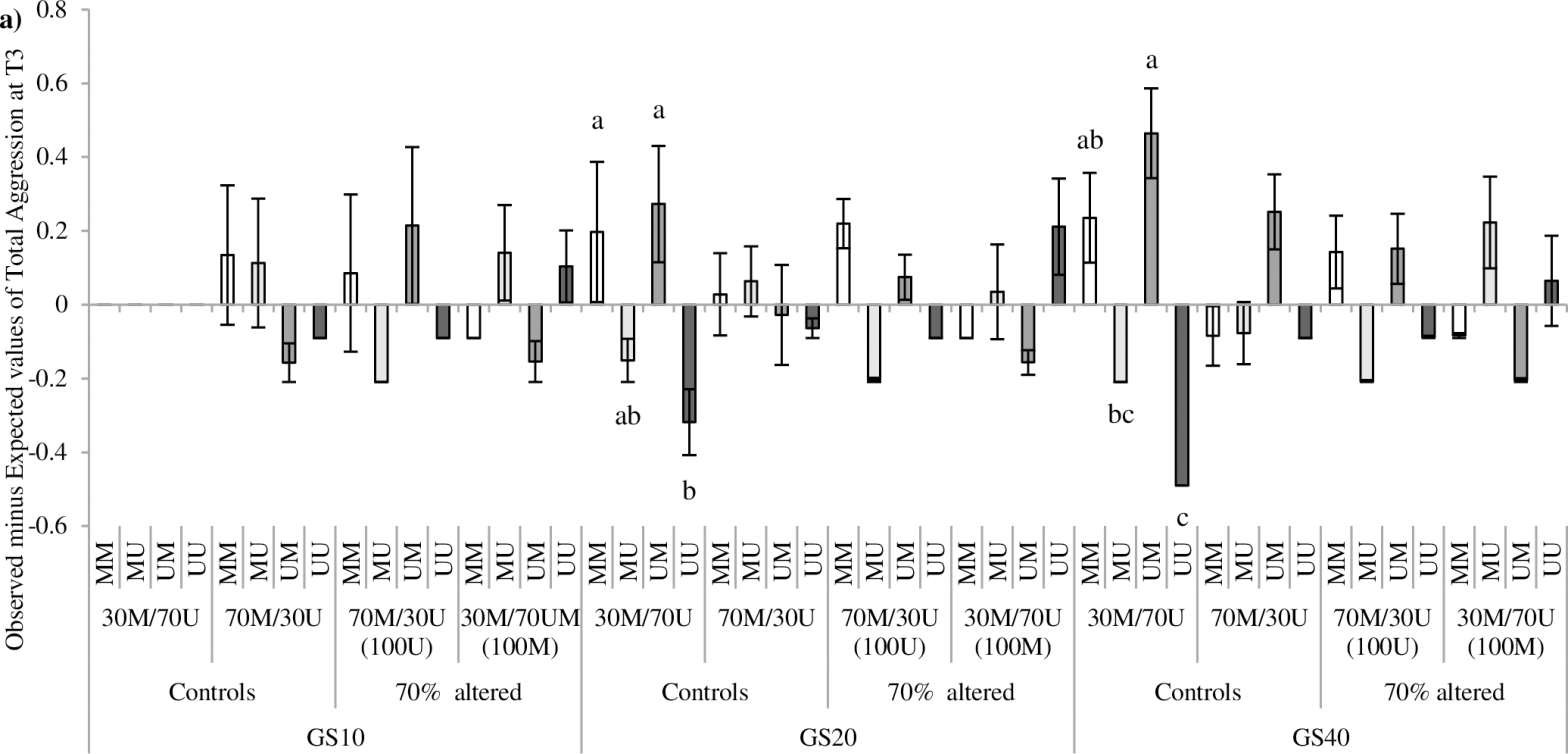
Directionality of aggressive interactions at T3. M = marked; U = unmarked. Differences between observed and expected aggressive interactions (means ± SE) at T3 for each possible interacting pair (MM, MU, UM and UU) and phenotypic appearance (PA) treatment: originally homogeneous (100U, 100M) and controls (30M/70U, 50M/50U, 70M/30U), according to group size (GS 10, 20 or 40 birds). Different letters indicate significant differences, within each GS and PA treatment, for each type of interacting pair (*P*<0.05).

A clear directionality in the occurrence of aggressive interactions, for each possible interacting pair (MM, MU, UM, UU), was found (PA by interacting pair at T0: *F*_6,64_ = 8.95, *P* <0.0001, Fig 3A; T1: *F*_9,88_ = 51.43, *P* <0.0001, Fig 3B; T2: *F*_6,64_ = 19.69, *P* <0.0001, Fig 3C; and T3: *F*_9,88_ = 12.76, *P* <0.0001, Fig 3D). Evidences of directionality were observed at T0 in 30M/70U control groups (Fig 3A). Clear directionality of aggression was also found at T1 in 30M/70U recently altered groups and controls (Fig 3B), with higher than expected interactions from U towards M birds and lower than expected interactions from M towards U birds. Conversely, 70M/30U recently altered groups at T1, presented clear directionality of aggression form M to U birds (Fig 3B). Regarding 50M/50U recently altered groups at T2, strong directionality of aggression towards the new emerging phenotypes was also reported: from U towards M birds (initially 100U groups) and from M towards U, (initially 100M; Fig 3C). A similar but somehow reduced directionality of aggression was observed at T3 (Fig 3D). A significant effect of the interaction between GS and PA was detected at T3 (*F*_18,88_ = 2.53, *P*<0.01; Fig 4). The clearest differences on directionality of aggression were observed in 30M/70U control groups at GS 20 and GS 40. No other evidence of directionality was found for either GS 10, 70M/30U control or recently altered groups.

## Discussion

The purpose of this study was to determine the effects of sequentially altering the phenotypic appearance (PA) of adult laying hens reared in originally homogeneous groups (100M or 100U) at three different GS (10, 20 and 40). The results of this work showed that the frequency of aggressive interactions was low and similar across all GS and original PA treatments at the onset of the study (T0, Fig 1A). Conversely, a substantial increase in aggression was observed at T1 after the 1^st^ sequential PA change was introduced (30% of hens altered in 100U or 100M groups; Fig 1B).

Previous studies have shown that domestic fowl can discriminate among group members [48], have preferences to stay close to familiar individuals [49–50] and show aggression to unfamiliar individuals [22, 50–54]. Other studies have also indicated that familiar birds with modified feathers or combs were targeted for aggression when reintroduced in the group [25, 26, 55, 56]. This was interpreted as evidence of the birds´ ability to discriminate between familiar and unfamiliar individuals. However, the sharp increase in aggression observed following alteration of the original phenotypes (for all GS) indicates that laying hens responded intensely escalating aggression to the emergence of new phenotypes. Furthermore, this effect was not mitigated by the potential capacity for individual recognition assumed in small groups. This was unexpected considering that the only change applied was to the colour of feathers on the back of the head.

Studies conducted in young meat and laying strains of domestic fowl [32, 45], and other animal species (reviewed by Murray and Fuller [57]) evidenced that marking can affect health, performance and behavior, due to social factors or to added difficulties to carry out normal activities. This study explored the process further, showing that similar effects occur not only when a new mark is added (marking 100U), but also when an existing mark is removed (unmarking 100M). To our knowledge, this is the first time this phenomenon has been investigated.

Dennis et al. [32] proposed four possible mechanisms to explain targeted aggression towards birds with altered phenotypes: 1) fear due to novelty of the marks, 2) xenophobia based on phenotypic dissimilarity, 3) marks perceived as signals of status, and 4) social challenge to conspicuous individuals. Mechanisms 1, 3 and 4 imply that the phenotype of altered individuals is conspicuous as a result of the new dark coloration used. However, our study showed that altered individuals, with or without a dark mark, attracted aggression at similar statistical levels. Thus, our current findings do not support the proposed mechanisms of increased aggression described by mechanisms 3 or 4. We interpret these results as evidence that the change in the phenotypic appearance itself is what caused the escalation in aggression, regardless of GS.

Another possible explanation for our results would involve phenotype matching mechanisms. Phenotype matching is used by animals to learn the phenotypes of their group-mates, creating a template to compare against phenotypes of new, unfamiliar individuals [39, 40]. Phenotype templates are normally shaped by imprinting during the first weeks of age [40, 58, 59]. This short time period is linked to the close proximity of chicks with parents and siblings, which ensures the correct development of phenotype templates. This is essential for survival and fitness, as it will ensure correct species identification for reproduction and recognition of potential competitors for resources. The sudden raise in aggression caused by the PA alteration to homogeneous groups could have been expected in large groups where individual recognition was unlikely [10, 18]. However, the lack of GS effects indicated otherwise. PA alteration severely affected aggression even at GS 10, where stable social structures based on individual recognition would have been expected after 33 weeks of cohabitation. These results evidence the birds´ inflexibility to accept new phenotypes once a template has been established. This strong response towards altered phenotypes could be indicating the high impact that ´invaders´ may have had in local populations through their evolutionary history. Invasions of the local populations by unrelated individuals, likely differing in phenotype, may have increased their exposure to new pathogens or parasites [41, 60, 61] and increased the competition for resources or during mating [38, 62]. Aggressively excluding these unrelated phenotypes would have been the most advantageous strategy to ensure the survival of the local population.

Adding a higher proportion of altered birds at T2 (50M/50U, Fig 1C) did not have as much impact. Although the level of aggressive interactions was still significantly higher than for control groups, the interactions started declining to reach almost basal levels by T3 (Fig 1D). The progressive reduction on aggression as the proportion of altered individuals increased suggests that, despite the social turmoil, birds were able to adapt and incorporate the emerging phenotype into their acceptable templates. Nevertheless, it is important to indicate that in wild populations, a similar escalation in aggression would have been, most likely, sufficient to force birds carrying the new phenotype to leave the group. In this study however, the restriction of the confined environment may have induced the acceptance of the new phenotype over time.

The decline in aggressive interactions could also be explained by a diluting effect [47]. If birds with altered phenotypes were targeted, then a diluting effect of directed aggression may be occurring as more birds would share the ´cost´ of carrying the new phenotype and fewer original birds will initiate the attacks. It can be argued that, perhaps, a similar decline may have occurred naturally if the groups were left with 30% of altered birds over an extended period of time. This is certainly a likely possibility and the study would have benefited from the inclusion of control pens to test this possibility. However, it was unfeasible to add any further treatments to this large experiment as the facilities were fully occupied.

Despite this shortfall, the strong directionality of the aggressive interactions towards the newer phenotypes revealed by this study is a relevant finding. Aggressive interactions were initiated at a higher than expected rate by individuals from the original and most frequent phenotype, and were clearly directed towards recently altered birds (Fig 3B and 3C). In 30M/70U groups (controls and recently altered; T1) aggression was directed from U towards M birds, while in 70M/30U (controls and recently altered; T1) the direction was from M towards U birds (Fig 3B). A similar pattern was observed in 50M/50U groups at T2, even when proportions of each phenotype within the pens were identical (Fig 3C). In addition, lower than expected interactions took place among the most common phenotypes in the groups; UU in 30M/70U (control and recently altered) and MM for 70M/30U (control and recently altered). Previous studies suggested that individuals that look different because of dull or soiled plumage colorations could be considered carriers of transmittable pathogens [63–64] and would probably be pushed away from the group. The strong directionality of aggression observed in our study may indicate that unaltered birds were trying to avoid the associated risk of living with unknown phenotypes that could lead to fitness costs. Thus, it is possible that natural factors producing changes in bird appearance, such as injuries, disease or feather pecking, may cause a similar reaction in healthy birds.

The current experiment demonstrates that the directionality of aggression towards altered phenotypes is equally remarkable when emerging as a consequence of adding new dark mark to the feathers, or by removing original dark marks from them. It does appear that the effects of marking were slightly stronger than unmarking birds but differences did not reach statistical significant levels. Even though the frequency of aggressive interactions after introducing phenotypic alterations was high, the large number of treatments may have diluted the potential differences between introducing ´new conspicuous phenotypes´ versus ´new dull phenotypes´. These potential differences should be further explored. However, increased aggression after experimental manipulation of badges of status has been observed in pukekos (*Porphyrio porphyrio melanotus)* [65]. These results were explained by ‘signal incongruence’, a mismatch between signal and behaviour, which leads to the animals’ attempts at reassessing the accuracy of the signal [66]. This same mechanism has been argued to explain the despotic behaviour observed towards sick animals [60, 67]. Birds in our study may have been able to detect a mismatch between their pen mates’ appearance and their behaviour. However, altered individuals in each pen were selected at random so a broad representation of social status among altered birds could be assumed. Initial determination of social status was not possible due to the large numbers involved (1050 birds) and the low level of aggressive interactions observed. In any case, both signal incongruence and phenotype matching mechanisms could explain our results as both should produce a similar response increasing aggression towards altered birds.

Despite the low frequency of aggressive interactions observed at T0, directionality was also observed from U towards M birds in 30M/70U control groups (Fig 1A). No directionality was detected for 50M/50U or 70M/30U. It could be argued that when phenotypic templates are established at an early age, only the most frequent phenotype would prevail as template for species recognition, by imprinting [68] or other mechanisms [36]. In this case, the opposite directionality of aggression would be expected towards unmarked birds in 70M/30U which were no detected at T0. However, after the first PA change at T1, directionality of aggression was observed in control groups from U towards M birds in the case of 50M/50U groups (at T2) and from M towards U in the case of 70M/30U groups (at T1). We speculate that the social instability created by the PA change may have somehow affected the house environment as a whole (i.e. auditory communication), disturbing control pens even if visual contact across treatment pens was not possible. It could be conceivable that under stress even control birds may have increased aggression levels towards individuals with the least frequent phenotypes. Given the age of the birds and the low levels of aggression observed at T0 in all groups, it could be assumed that the social structure, either based in a classic hierarchical system [1–3] or by the adoption of a tolerant system [10, 14], was stable. Despite this, the emergence of new phenotypes clearly triggered a social turmoil with a large increment in the frequency of aggressive interactions. We wrongly predicted increased aggression to be of higher relevance in larger groups. However GS effects were only observed at T3, when the frequency of aggressive interactions was returning to basal values. Our results provide strong evidences that PA alterations have in fact a much higher relevance than those of GS under the described conditions.

In conclusion, this study provides evidence that the emergence of new phenotypes in originally homogeneous groups of domestic fowl produces an escalation of aggression clearly directed towards birds presenting those new phenotypes. Interestingly, directionality of aggression was equally observed when birds were marked in a homogeneous unmarked population (increasing conspicuosity), and when birds were unmarked in a homogeneous marked population. Therefore, we rejected the status signalling hypothesis as a possible explanation to the effects of phenotype alteration. Phenotype matching mechanisms should be considered as a more parsimonious explanation to the reaction to new phenotypes that we observed. A cost effective strategy to reduce the risk of competition for resources, or health threats, to the local population from unfamiliar phenotypes should also be factored in. Even though the existence of a phenotypic template could explain the increase on aggression and its clear directionality observed towards altered birds, this may not be a static process. In the case of captive populations, such as farmed birds, the new phenotypes may also be integrated as part of their normal diversity over time, but the process may cause severe stress to the birds until they get acquainted with each other. Only marginal effects of GS were detected, suggesting that the impact of PA was far more important to grant social stability. These findings provide evidence that a simple mechanism, such as phenotype matching, could explain how populations deal and respond to varying phenotypic diversity. Phenotype matching may explain why diversity due to growth, injuries or diseases, could lead to escalation in aggressive interactions that may compromise the survival of the targeted birds.

## Supporting information

S1 Fig Photographic description of marked (M) and unmarked (U) birds participating on the current experiment. Marked (M) adult hen on the left and unmarked (U) adult hen on the right.
(PPT)
