# Supplementary figures and images for: The looks matter; aggression escalation from changes on phenotypic appearance in the domestic fowl
Source: PLoS One. 2017 Dec 20;12(12):e0188931. doi: 10.1371/journal.pone.0188931 (PMC5737892; doi:10.1371/journal.pone.0188931)

## Slide 1
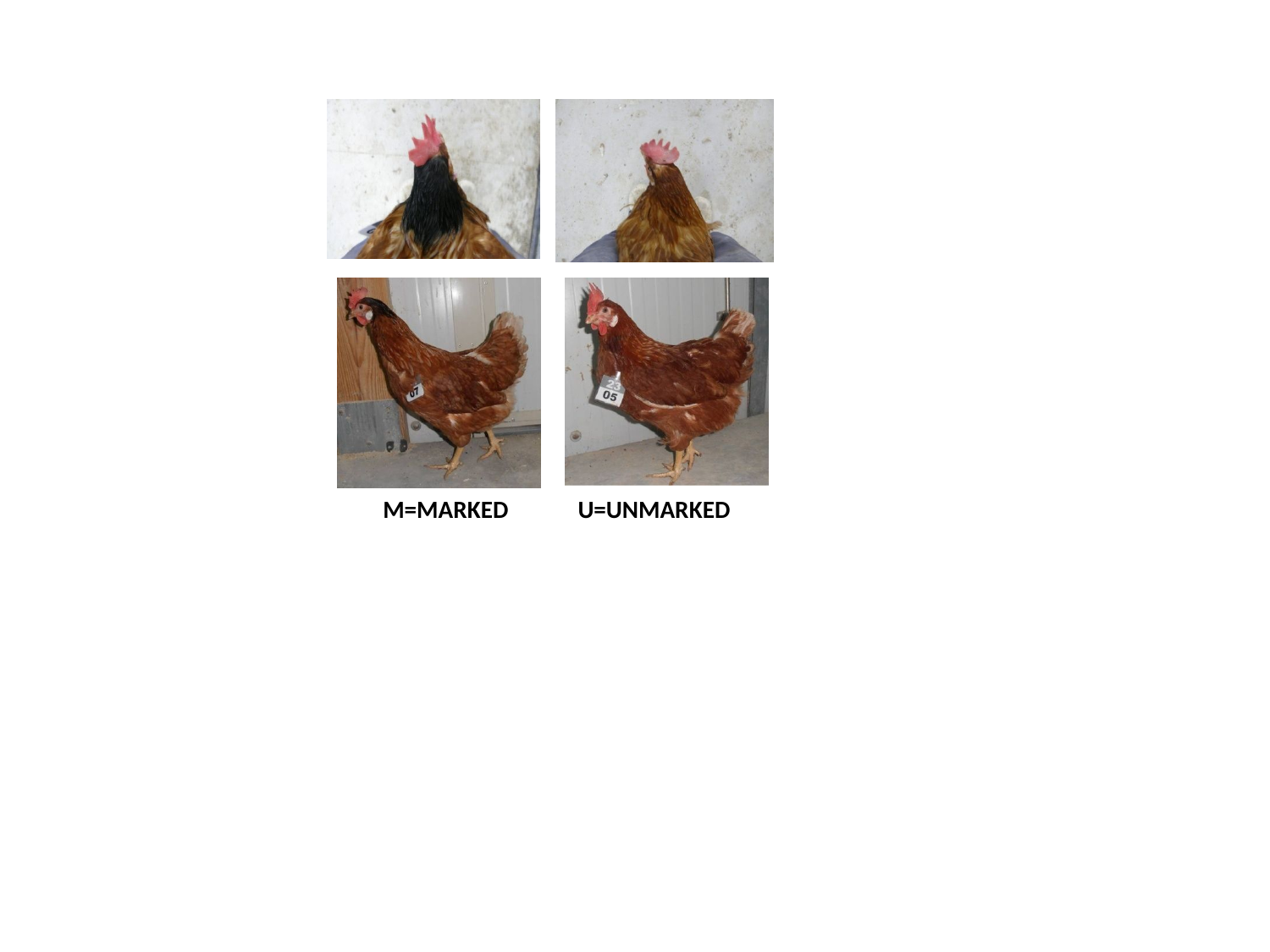

M=MARKED
U=UNMARKED

Supplement: S1 Fig — Marked (M) adult hen on the left and unmarked (U) adult hen on the right. (PPT) [file pone.0188931.s001.ppt]
